# Editorial: Nomenclature - Avoiding Babylonian Speech Confusion in Present Day Immunology

**DOI:** 10.3389/fimmu.2020.621100

**Published:** 2020-12-14

**Authors:** Menno C. van Zelm, Loems Ziegler-Heitbrock, Andrew M. Collins, Sanny K. Chan, Pablo Engel

**Affiliations:** ^1^ Department of Immunology and Pathology, Central Clinical School, Monash University, Melbourne, VIC, Australia; ^2^ Department of Allergy, Immunology & Respiratory Medicine, Central Clinical School, Monash University, Melbourne, VIC, Australia; ^3^ Allergy, Asthma and Clinical Immunology, Alfred Health, Melbourne, IC, Australia; ^4^ Monocytomics Research, Munich, Germany; ^5^ School of Biotechnology and Biomolecular Sciences, University of New South Wales, Sydney, NSW, Australia; ^6^ Department of Pediatrics, Division of Allergy and Immunology, National Jewish Health, Denver, CO, United States; ^7^ Department of Biomedical Research, National Jewish Health, Denver, CO, United States; ^8^ Immunology Unit, Department of Biomedical Sciences, Medical School of the University of Barcelona, Barcelona, Spain

**Keywords:** B cell, clusters of differentiation marker, allergen, cytokine, complement, C-type lectin receptor, immunoglobulin gene, monocytes and dendritic cells

## Introduction

The complexity of the immune system at the gene, protein, cell, and organism levels continues to provide a major challenge. Genomic landscaping, single-cell analysis and mass data acquisition including genome, transcriptome, metabolome, and proteome have now added new levels of complexity. With the rapid progress in these and other fields of immunology, it has become more important than ever to agree on uniform nomenclatures, i.e. to agree on how to name novel genes, proteins, cells, and biological reagents.

Names given initially might, in retrospect, not always be logical. For example, tumor necrosis factor (TNF) was named on the basis of the observation of central necrosis in an experimental subcutaneous mouse tumor model (1). It was only after many unsuccessful studies in cancer, that eventually the role of TNF as a master cytokine in inflammation emerged. By that time, it was too late to rename the molecule because that would cause renewed confusion.

Another cytokine has been successfully renamed. Interleukin-6 was initially known as B-cell Stimulatory Factor 2, Cytotoxic T lymphocyte Differentiation Factor, Hybridoma Growth Factor, Hepatocyte Stimulating Factor, and Interferon Beta-2. Obviously, such usage of different names for the same item can lead to confusion and may hinder progress in the field. These two examples demonstrate the need for a consensus nomenclature, which is timely applied.

Indeed, the impacts of early consensus nomenclature are enormous, and the immunological community has an excellent track record of conducting worldwide cooperative efforts on nomenclature issues (2). Remarkable examples of these include nomenclature for antigen receptor (IG and TR) genes (3), cytokines and chemokines and their receptors (4), as well as allergens (5), cell types (6, 7) and the CD nomenclature for monoclonal antibodies (8).

More recently, nomenclature initiatives agreed on an early consensus regarding classification of leukocytes. The monocyte nomenclature proposal in 2010 defined the population of “intermediate monocytes” (7), resulting in >2000 returns for this term under Google Scholar. Furthermore, consensus nomenclature has been defined for innate lymphoid cell (ILC) subsets (6), which will undoubtedly drive discoveries into their roles in tissue homeostasis, morphogenesis, metabolism, repair, and regeneration.

With the many achievements reached in the past 40 decades, there is a wealth of experience to draw upon, especially within the subcommittees of the Nomenclature Committee of the International Union of Immunological Societies (IUIS; https://iuis.org/). The IUIS Nomenclature Committee is fostering nomenclature efforts by providing a platform that currently includes the activities of altogether 11 nomenclature subcommittees (https://iuis.org/committees/nom/). Each subcommittee consists of a representative group of experts in the field, independently decides on nomenclature on the basis of consensus, and is typically endorsed by IUIS and one or more sister societies (e.g. Allergens by AAAI/EAACI/IUIS, Complement by ICS/IUIS).

The present Research Topic aims to highlight the need to address controversies and to stress the importance of nomenclature based on consensus within the immunology community. Twelve articles are included in this Research Topic, and are categorized into the following types: two Original Research (Kalina et al; Magadan et al.), four Reviews (Gunther et al.; Heger et al.; Ohlin et al.; Sanz et al.), two Mini Reviews (Chan et al.; Del Fresno et al.), two Opinions (Hsiao et al.; Zlotnik), and two Perspectives (Bohlson et al.; Busse et al.). The contributions are briefly covered below with the subtopics soluble mediators, cell surface receptors, immunoglobulin genes, cells of the immune system and allergens.

## Soluble Mediators

The nomenclature of complement components dates back to the 1960s (9), followed by a formalized nomenclature of the later discovered alternative pathway in 1981 (10). In 2014, an updated nomenclature of these components with inclusion of newly-identified receptors was published (11). Despite these collaborative efforts, in this issue Bohlson et al. identify several unresolved naming issues. Most importantly, in their present proposal, the nomenclature for the cleavage fragments of C2 is brought into line with all other components (C3, C4, C5, Factor B), such that the smaller fragment is now designated “C2a”and the larger fragment “C2b”. Additional updates to clusterin, C1 complex activation states recognition molecules (PRMs) and enzymes of the lectin pathway and regulatory proteins of the complement system are proposed as an update to the 2014 Nomenclature.

Nomenclature of soluble mediators of the immune system has been a major challenge, and this was recognized already in the 1970s, resulting in the consensus nomenclature of IL-1 and IL-2. Despite such activities and structured naming of cytokines, chemokines and their receptors, Zlotnik identifies several current issues and challenges. These include the ‘neutral’ nomenclature of interleukins, which does not relate to their (inflammatory or anti-inflammatory) biological activity, or their evolutionary relationships. Furthermore, the CXC and CC chemokines are well-defined, but challenges have arisen for more recently-identified chemokines that are located in other genomic regions. Thus, in this field there are ongoing nomenclature challenges, which require clear and perhaps updated definitions of what would qualify as a novel cytokine and/or chemokine.

## Cell Surface Receptors

Since the 1980s, Human Leukocyte Differentiation Antigen (HLDA) workshops have been organized to test and name clusters of antibodies that reacted with a specific antigen (8). These cluster of differentiation (CD) markers provided consistency and uniformity in manuscripts when referring to identical molecules. CD markers have proven critical for the identification and isolation of leukocytes and lymphocyte subsets, the diagnosis and follow-up of hematological malignancies, autoimmune diseases, and immunodeficiencies, and the monitoring of cancer immunotherapy. However, there are important gaps in our knowledge of CD molecule expression profiles, especially because of the major advances in multiparametric flow cytometry over the last 30 years. The paper by Kalina et al. presents a pilot study that shows the expression patterns of CD1 to CD100 on 47 leukocyte subsets from the blood, thymus and tonsil, using highly standardized eight-color flow cytometry. The resulting dataset includes median antibody binding capacities and percentage of positivity for all markers on all subsets, and can be explored online through an interactive CD Maps web resource (www.hcdm.org). The data presented in this paper will provide a better picture of the surfaceome of human leukocytes and increase our understanding of leukocyte biology.

The Ca++-dependent type lectin receptors (C-type lectin receptors; CLRs) offer an example for Babylonian speech confusion. Del Fresno et al. explain that there can be up to seven different names for a single CLR, and that the same name is used for different CLRs between man and mouse. Here they analyzed the frequency of use of the different names in the literature. They suggest the gene name be mentioned for a given CLR plus the most frequently used name in the abstract of every paper on the topic. This recommendation can help to overcome the nomenclature confusion in the field.

A nomenclature for adhesion G protein–coupled receptors (ADGR) was published several years ago (12). In this nomenclature the brain-specific angiogenesis inhibitor 1 (BAI1) has been renamed ADGRB1. For this receptor, expression in macrophages had been reported in 2007. Hsiao et al. revisit this point and they have extracted data on ADGRs from proteome and transcriptome repositories. None of the available data sets contained a signal for ADGRB1 in monocytes/macrophages and this included RNAseq analyses, which can pick up low abundance transcripts. The study contributes to clarification of an important issue in the field of ADGRs.

## Immunoglobulin Genes

Previously-unreported *IGHV* alleles are often a conspicuous presence in human datasets of rearranged VDJ gene sequences (13, 14), but there has been no mechanism by which they can be named. With support from the ImMunoGeneTics (IMGT) Group, Ohlin et al. describe processes that can now lead to the official naming of such sequences.

Magadan et al. describe a new nomenclature to deal with the complexities of the *IGH* loci of salmonid species. This task is made particularly challenging by the fact that the *IGH* loci of salmonid species are duplicated on separate chromosomes, and both loci can rearrange to form functional VDJ genes. In this study, genomic assemblies of the *IGH* loci of the Atlantic salmon and Rainbow trout have been annotated, and IG genes have been named according to the IMGT positional-within-locus nomenclature rules.

Busse et al. address the nomenclature challenges that arise from structural variation in the *IGH* loci of laboratory mice. The IMGT positional-within-families mouse *IGH* nomenclature is based upon annotations of the *IGH* locus of the C57BL/6 mouse genome reference sequence, but this sequence is remarkably different to the *IGH* loci of other inbred strains (15, 16). Busse and colleagues outline the principles that should guide the development of a new nomenclature to deal with this challenge. They argue in favor of a non-positional nomenclature, for this would facilitate the naming of hundreds of mouse *IGHV* genes that are now known, but which remain unmapped and unnamed. Non-positional nomenclature would also avoid the need for the renaming of some IGHV sequences, when new genome assemblies identify errors in previous gene maps. Such changes have resulted in confusion within the research community in the past.

## Cells of the Immune System

With the availability of an increasing number of new markers, there is the temptation to define more and more cell subsets. While in the past a bimodal expression of a cell surface molecule on a given leukocyte was considered sufficient to define two subsets, we now require, in addition, a differential transcriptome, differences in function and, for consolidation, informative clinical associations.

The renewed attention to B cells during the last decade has resulted in the identification of many new subsets that are inconsistently defined and named. Thus, there is an urgent need for a consistent nomenclature of human B cells to allow for inter-laboratory interpretability. The very comprehensive review by Sanz et al. presents a unified approach of classification based on phenotypic standardization. The authors propose the use of seven surface markers, using multiparameter flow cytometry, to define a variety of functionally-distinct B-cell populations. They also discuss the need for awareness that not all current surface antigens being utilized for defining distinct B-cell subsets are sufficiently conclusive. This Perspective is meant to initiate a discussion in the B-cell community with the aim to reach an international consensus nomenclature for B cells.

The heterogeneity of monocytes and dendritic cells and the impact of extensive data sets is covered by Gunther et al.. The paper points out that many different myeloid cell types and subsets have been defined on the basis of morphology, cell surface marker expression and function. Much of this has been confirmed by mass cytometry, multicolor flow cytometry, and by single cell sequencing, but additional populations emerged. The pitfalls of these novel approaches, including misclassifications, are discussed and unbiased strategies for future research are presented.

The nomenclature of dendritic cells has been difficult because of a trend to name any cell “dendritic cell”, as long as it could induce a T cell response. More recently, a more stringent definition has been used with pDCs, DC1s and DC2s being considered *bona fide* DCs. With respect to DC2s, a detailed analysis has demonstrated there is a subset, which lacks typical DC features but instead shows markers and functions that are characteristic of monocytes/macrophages. Heger et al. review the latest developments in this area and discuss whether or not these cells belong to the monocyte lineage.

## Allergens

Since at least the early 1980s leading allergists started to standardize the naming process for protein allergens that cause IgE-mediated reactions in humans. The use of the taxonomic name of the source organism now ensures a consistent nomenclature that enables communication within allergy research and clinical care and with external regulatory bodies (17). Today, applying for a unique name from the WHO/IUIS Allergen Nomenclature Sub-Committee is a critical step prior to the publication of data on a novel allergen (5).

The paper by Chan et al. reviews the current procedures and requirements for the submission of new proteins allergens and the reasons behind recent changes. These changes are related to advances regarding a) the amount and route of exposure that causes a protein to become an allergen; b) the structural biology of allergen subunits and their contribution to larger complex allergen structures; c) non-protein allergens such as complex carbohydrates. This paper will be helpful to colleagues, who plan on submitting new allergens to the Allergen Nomenclature Committee.

Together, the articles in this Research Topic illustrate the ongoing need for active governance of existing and assignment of new nomenclatures. There is vast experience in the immunological research community to deal with such complex issues. The IUIS Nomenclature committee has a history of bringing leaders in the field together for timely and open discussion, and will remain committed to supporting current and new consensus nomenclature initiatives.

## Author Contributions

All authors contributed to the article and approved the submitted version.

## Funding

MZ is supported by the Australian National Health and Medical Research Council (NHMRC; Senior Research Fellowship 1117687) and the Jeffrey Modell Foundation.

## Conflict of Interest

The authors declare that the research was conducted in the absence of any commercial or financial relationships that could be construed as a potential conflict of interest.
